# Genome Sequence of a Newly Isolated Mycobacteriophage, ShedlockHolmes

**DOI:** 10.1128/genomeA.00597-15

**Published:** 2015-06-18

**Authors:** Welkin H. Pope, Jordan T. Carter, Kegan L. Dasher, Mikaela C. Haynberg, Anoop Reddi, Kathleen A. Shedlock, Jonathan S. Lapin, Ashley K. Prout, Sarah R. Grubb, Marcie H. Warner, Charles A. Bowman, Daniel A. Russell, Graham F. Hatfull

**Affiliations:** Department of Biological Sciences, University of Pittsburgh, Pittsburgh, Pennsylvania, USA

## Abstract

Mycobacteriophage ShedlockHolmes is a newly isolated phage infecting *Mycobacterium smegmatis* mc^2^155. It has a 61,081-bp genome containing 99 predicted protein-coding genes and one tRNA gene. ShedlockHolmes is closely related to mycobacteriophages Pixie, Keshu, and MacnCheese and is a new member of subcluster K3.

## GENOME ANNOUNCEMENT

The large collection of sequenced mycobacteriophages provides a resource for understanding viral diversity and evolution and is a means to develop systems for understanding tuberculosis genetics (1, 2). Mycobacteriophages are the largest group of sequenced phages known to infect a single common host (3) and can be readily isolated from environmental samples, like soil or compost, using *Mycobacterium smegmatis* mc^2^155 as a host (3). Freshman students participating in the Science Education Alliance Phage Hunters Advancing Genomics and Evolutionary Science (SEA-PHAGES), which exposes students to authentic research and scientific discovery, are responsible for isolating and analyzing these phages (4).

Mycobacteriophage ShedlockHolmes was isolated from a soil sample in Pittsburgh, PA, using *M. smegmatis* mc^2^155 as a host. Electron microscopy shows that ShedlockHolmes has a flexible noncontractile tail and an isometric head. It was purified and amplified, and its DNA was isolated and sequenced using an Illumina MiSeq instrument. Single-end run reads of 140 bp were assembled using Newbler to give a contig with 343-fold coverage. The ShedlockHolmes genome is 61,081 bp, and coverage analysis shows it has *cos* ends with 11-base 3′ extensions of the sequence 5′-CTCGATGGCAT. The G+C content of ShedlockHolmes is 67.3%, similar to that of its host, *M. smegmatis*.

Putative protein-coding genes in the ShedlockHolmes genome were identified by autoannotation using the heuristic models in Glimmer and GeneMark, followed by manual inspection and annotation revision. A total of 99 protein-coding genes were predicted, accounting for a 91.74% coding capacity of the genome. BLASTn analysis showed that ShedlockHolmes is closely related to mycobacteriophages Pixie, Keshu, and MacnCheese, all of which are in subcluster K3, and it joins them in this subcluster. The closest relative to ShedlockHolmes is Pixie, with which it shares 95% nucleotide identity across 98% of the genome span, although there are at least six syntenic interruptions due to insertions, deletions, or gene substitutions.

Like other cluster K phages, ShedlockHolmes has several features common to temperate phages. It encodes an integrase of the tyrosine-recombinase type and is predicted to integrate into an *attB* site located at a host tRNA^Lys^ gene (Msmeg_4746). ShedlockHolmes gp46 contains a putative helix-turn-helix DNA binding motif and is a good candidate for the phage repressor. We also identified 18 instances of a start-associated sequence (SAS) described in other cluster K phages (5). Fifteen of these correspond to the consensus sequence (5′-GGGATAGGAGCCC), one has a single-base difference, and two have two-base differences. With one exception, all of the SASs are closely linked to predicted translational start sites of genes in the right arm of the genome; their role in translation initiation is unknown.

Comparative analyses reveal the mosaic nature of ShedlockHolmes. For example, ShedlockHolmes *30* homologues are absent in all other cluster K genomes but are present in >60 genomes in subclusters A2 and A4. We also note that ShedlockHolmes gp26 is closely related to subcluster A3 tail proteins and is implicated in determining host range and the ability of A3 and K phages to infect *Mycobacterium tuberculosis* and *M. smegmatis*.

### Nucleotide sequence accession number.

The ShedlockHolmes genome sequence is available from GenBank under the accession no. KR080206.
